# On Stertor, Apoplexy, and the Management of the Apoplectic State

**Published:** 1891-09

**Authors:** 


*On Stertor, Apoplexy, and the Management of the Apoplectic State.* By ROBERT L. BOWLES, M.D., F.R.C.P. Pp. 125. London: BAILLIÈRE, TINDALL & COX. 1891.

Dr. Bowles's researches on this subject, which extend over a period of thirty years, are already widely known through the medium of the *Transactions* of various Societies, and of the *Journals*. In this book he has collected them together, and added some original observations on the structure and anatomical arrangements of the mammalian pharynx and epiglottis. Many useful directions for the management of apoplexy and the relief of stertor in the apoplectic state are given, and should be familiar to all medical men. The methods which he recommends for obviating stertor are simple, easily carried out, and would seem to be effectual; they are founded on a careful study of the mechanism of its production under different conditions. Everyone will agree with the author as to the importance of keeping the respiratory passages patent in apoplectic states, and that in most cases the exact diagnosis of the nature and position of the lesion would be greatly facilitated if stertor could be prevented. The further contention that when in apoplexy there is raised blood-pressure, a tense full pulse, and powerful action of the heart, these phenomena are in the large majority of cases caused by interference with the proper aëration of the blood through obstruction in the upper air-passages, we should not be prepared to admit without further evidence. The author's views as to the production of stertor are based on careful clinical observation and patient experimental research, and deserve to be widely known. We welcome this book as putting them in a convenient form.

